# Quinolone resistance phenotype and genetic characterization of *Salmonella enterica* serovar Pullorum isolates in China, during 2011 to 2016

**DOI:** 10.1186/s12866-018-1368-4

**Published:** 2018-12-27

**Authors:** Xiaodong Guo, Honglin Wang, Yiluo Cheng, Wenting Zhang, Qingping Luo, Guoyuan Wen, Guijun Wang, Huabin Shao, Tengfei Zhang

**Affiliations:** 1 Key Laboratory of Prevention and Control Agents for Animal Bacteriosis, Institute of Animal Husbandry and Veterinary, Hubei Academy of Agricultural Sciences, Wuhan, China; 2 College of Animal Science and Technology, Anhui Agricultural University, Hefei, China; 3 Hubei Key Laboratory of Animal Embryo and Molecular Breeding, Institute of Animal and Veterinary Science, Hubei Academy of Agricultural Sciences, Wuhan, China; 4 Hubei Engineering Technology Center of Veterinary Diagnostic products, Wuhan, 430070 China

**Keywords:** *Salmonella enterica* serovar Pullorum, Chicken, MLST, Quinolone resistance, Biofilm

## Abstract

**Background:**

Pullorum disease, caused by *Salmonella enterica* serovar Pullorum (*S*. Pullorum), is one of the most important bacterial infections in the poultry industry in developing countries, including China. To examine the prevalence and characteristics of *S*. Pullorum, the Multilocus Sequence Typing (MLST) genotypes, fluoroquinolones resistance, and biofilm-forming abilities of *S*. Pullorum isolates were investigated, collected from 2011 to 2016 in China.

**Results:**

Thirty *S*. Pullorum isolates collected from 2011 to 2016 were analyzed. Quinolones susceptibility testing showed that 90% of the isolates were resistant to the first generation of quinolines nalidixic acid, but the resistance rates to different fluoroquinolones agents were lower than 13.3%; for some there was even no resistance. Multilocus sequence typing (MLST) showed that ST-92 was the dominating genotype, accounting for 90.0% of all *S*. pullorum strains. The remaining three isolates were of the new reported sequence type ST-2151. Interestingly, the Asp87Gly substitution in quinolone resistance-determining regions (QRDR) of GyrA was only observed in the three strains of ST-2151, suggesting a potential correlation between Asp87Gly substitution and sequence type (*p* < 0.05). However, Asp87Gly substitution could not confer the resistant to ofloxacin and ciprofloxacin of these isolates. The plasmid-mediated quinolone resistance (PMQR) gene was not found in any of the tested isolates. Furthermore, an assay measuring biofilm-forming abilities showed that 46.7% of the isolates were non-biofilm producers, while 53.3% could form very weak biofilms, which might explain the relatively lower resistance to fluoroquinolones.

**Conclusions:**

We reported a high resistance rate to the first generation of quinolines nalidixic acid and relatively low resistance rates to fluoroquinolones in *S*. Pullorum isolates. In addition, weak biofilm-forming abilities were found, which might be an important reason of the low fluoroquinolones resistance rates of *S*. Pullorum isolates. ST-92 was the dominating genotype demonstrated by MLST, and the new sequence type ST-2151 showed a potential correlation with Asp87Gly substitution in QRDR of GyrA. We believe the characterization of these *S*. Pullorum isolates will be helpful to develop prevention and control strategies.

**Electronic supplementary material:**

The online version of this article (10.1186/s12866-018-1368-4) contains supplementary material, which is available to authorized users.

## Background

*Salmonella enterica* serovar Pullorum (*S*. Pullorum) can cause severe infectious pullorum disease (PD) in chicken and some other domestic birds, leading to a serious threat to the poultry industry [1]. Because the transmission of *S*. Pullorum occurs both horizontally and vertically [2], eradication programs, especially for breeding birds, are carried out in many countries. However, due to extensive testing and eradication costs, in addition to the genetic diversity of *S*. Pullorum, *S*. Pullorum is still very common in the poultry industry in Africa and Asia, including China [3, 4].

In most of the developed countries, strict eradication programs have eliminated *S*. Pullorum from the commercial poultry flocks [5], but in noncommercial poultry flocks, outbreaks of PD occur constantly [6]. In China, *S*. Pullorum is still a widespread pathogen in the poultry industry. Gong et al. found that, from 2006 to 2012, *S*. Pullorum was the most frequent serovar of *S. enterica*, accounting for 17.0% [7]. Liu et al. reported that 17 out of 121 *Salmonella* strains obtained from food, fodder and live chickens were *S*. Pullorum [8]. Investigating the genetic characterization of epidemic strains will help us to better understand the epidemiology. Multilocus sequence typing (MLST) has been used to study the evolution and epidemiology of a number of bacterial pathogens, with the advantage of comparing the results across various laboratories using the same analysis [9].

In addition to eradication, use of antimicrobial drugs is still one of the main measures to control *S.* Pullorum infection. However, the use of antimicrobial agents contributes to the dissemination of antimicrobial resistance, and results in an increase of multiple drug-resistant bacteria [10]. Therefore, use of chloramphenicol, tetracycline and some other older antimicrobials is now limited in animal feeding, and fluoroquinolones are one of the most commonly used antimicrobial agents in poultry farming. Unfortunately, with the use of fluoroquinolones, resistant *Salmonella* strains are increasing worldwide [11–13]. The main resistance mechanisms include mutations in quinolone resistance-determining regions (QRDR) of DNA gyrase and topoisomerase [14], and presence of a series of plasmid-mediated quinolone resistance (PMQR) genes [15]. Mutations in QRDRs and presence of PMQR have been frequently reported in foodborne *Salmonella* infections in China and other countries [16, 17].

To examine the prevalence and characteristics of *S*. Pullorum, the MLST genotypes, fluoroquinolones resistance, and biofilm-forming abilities of *S*. Pullorum isolates were investigated, collected from 2011 to 2016 in China.

## Methods

### Isolation and identification

From 2011 to 2016, 692 swab samples were collected from chickens with PD and healthy-looking chickens in five provinces of China. Chickens with PD showed typical symptoms, such as white diarrhea, lethargy and so on, and some were dead. Healthy-looking chickens were chickens without obvious symptoms. Collected samples were directly placed into Cary-Blair modified transport media (AMRESCO, USA) and transported to the laboratory for *Salmonella* isolation. Swabs were cultured in 9 mL of Gram negative (GN) broth (Tianhe, China) at 37 °C for 24 h before aliquots of 100 mL of the broth were streaked onto Triple Sugar Iron agar (TSI, Oxoid, England). Typical *Salmonella* colonies were confirmed by PCR amplification of the *hut* gene, the primers were as followed, hut-F, 5’-ATGTTGTCCTGCCCCTGGTAAGAGA-3′, and hut-R, 5’-ACTGGCGTTATCCCTTTCTCTGCTG-3′ [18]. *S*. Gallinarum biovar Pullorum was determined by a slide agglutination test with O-antigen antiserum and a tube agglutination test with H-antigen antiserum [19]. The isolates were also identified using duplex PCR analysis, as described previously [20].

### MLST

MLST was carried out to determine the genetic diversity of the isolates, as previously described [21]. Briefly, seven housekeeping genes (*aroC*, *dnaN*, *hemD*, *hisD*, *purE*, *sucA,* and *thrA*) in each tested isolate were amplified and sequenced. Sequences alignment were carried out using the *Salmonella enterica* MLST database (http://enterobase.warwick.ac.uk/species/senterica/allele_st_search), and allele numbers and sequence types (STs) were assigned.

### Biofilm-forming abilities assay

Biofilm formation abilities were assessed by crystal violet staining as previously described [22, 23]. Briefly, 100 μL of the cell culture (OD_590nm_ = 0.1) was added to a 96-well polystyrene tissue culture plate (Corning, USA) and incubated at 37 °C for 48 h to form biofilms. To stain with crystal violet, cells were discarded, and each well was washed with water and dried. Then, 120 μL of 1% crystal violet solution was added and incubated without shaking for 30 min at room temperature. After washing off the unbound crystal viole, bound crystal violet was dissolved in 20% (*v*/v) acetone-containing ethanol and the OD_630nm_ of the dissolved crystal violet solution was measured. All the tests were performed in triplicate. The *Salmonella* Enteritidis CVCC3375 strain was used as a positive control and three wells without inoculated bacteria were used as the negative control. Two times the negative control value was defined as the cutoff OD value (ODc) [24, 25]. According to the OD values, strains were classified into non-biofilm producer (OD ≤ ODc), weak biofilm producer (ODc < OD ≤ 2 × ODc) or strong biofilm producer (OD > 2 × ODc) [24, 25].

### Quinolones susceptibility testing

According to the Clinical and Laboratory Standards Institute Standards guidelines (CLSI) [26], (fluoro)quinolones susceptibility of the *S*. Pullorum isolates was determined by the disk diffusion method as previously described [23]. A total of five (fluoro)quinolones antibiotics, including ciprofloxacin, ofloxacin, enrofloxacin, norfloxacin, and nalidixic acid (Oxoid, England) were tested. *E. coli* strain ATCC 25922 was used as the quality control.

### Detection of mutations in QRDR and PMQR

To detect mutations in QRDRs of DNA gyrase and topoisomerase in the isolates, DNA was isolated and four genes of each strain, including *gyrA*, *gyrB*, *parC,* and *parE*, were amplified by PCR, as previously described [27]. The products were sequenced and mutations in QRDRs were identified by sequence alignment. To detect mutation in the PMDR genes, *AAC-Ib*, *qnrA*, *qnrB*, *qnrC*, *qnrD*, *qnrS*, integrase, and intergron were amplified by PCR using the primers and amplification conditions as previously described [28].

### Statistical analysis

To test for the correlation in resistance rates and biofilm-forming abilities between different sources, and the correlation between point mutation and sequence type, the fisher test was performed with *p* < 0.05 considered statistically significant. The statistical analysis software was SPSS 19.0.

## Results

### Identification and MLST of *S.* Pullorum

A total of 30 *Salmonella* strains collected from 2011 to 2016 were identified as *S.* Pullorum. The isolates are listed in Table 1. Of these strains, 18 were isolated from chicken with PD, and 12 were isolated from chickens without obvious symptoms.

**Table 1:** *S*. Pullorum isolates in this study

Strains	Susceptibility to different (fluoro)quinolones agents^a^					Mutation in DNA gyrases		Sequence types	Biofilm-forming ability^b^	Province (year)	Sources^c^
	CIP	OFL	ENR	NOR	NA	GyrA	GyrB				
TC03	S	S	S	S	R			ST92	–	Anhui (2011)	spleen of DC
TC07	S	S	S	S	R			ST92	–	Anhui (2012)	liver of DC
KQ58	S	S	I	S	R			ST92	–	Anhui (2012)	cecum of DC
HF60	S	S	I	S	R			ST92	+	Anhui (2015)	anus swabs of DC
WX46	S	S	I	I	R	Asp-87-Gly	Leu-451-Ile	ST2151	–	Anhui (2016)	spleen of DC
WX47	S	S	I	I	R	Asp-87-Gly		ST2151	+	Anhui (2016)	spleen of DC
XY83	S	S	I	R	R			ST92	+	Henan (2013)	cecum of DC
XY84	S	S	S	S	S			ST92	–	Henan (2013)	cecum of DC
WH59	S	S	I	I	R	Asp-87-Gly		ST2151	–	Hubei (2013)	anus swabs of DC
YZ01	S	S	S	I	R			ST92	+	Jiangsu (2011)	liver of DC
JD02	S	S	I	I	R			ST92	+	Jiangsu (2011)	liver of DC
JD04	S	S	R	S	R			ST92	–	Jiangsu (2012)	liver of DC
HA05	S	S	S	S	R			ST92	–	Jiangsu (2012)	liver of DC
YC06	S	S	S	S	R			ST92	–	Jiangsu (2012)	ovary of DC
JD11	S	S	S	I	R			ST92	+	Jiangsu (2014)	liver of DC
YZ12	S	S	I	R	R			ST92	+	Jiangsu (2014)	liver of DC
QD14	S	S	S	I	R			ST92	–	Shandong (2014)	cecum of DC
RZ15	S	S	I	S	R			ST92	+	Shandong (2015)	cecum of DC
WH72	S	S	S	S	R			ST92	–	Hubei (2013)	anus swabs of HLC
WH73	S	S	S	R	R			ST92	+	Hubei (2013)	anus swabs of HLC
WH74	S	S	S	R	R			ST92	+	Hubei (2013)	anus swabs of HLC
HS77	S	S	S	I	R			ST92	–	Hubei (2013)	anus swabs of HLC
GC80	I	S	I	S	R			ST92	+	Hubei (2015)	anus swabs of HLC
GC81	S	S	S	S	S			ST92	–	Hubei (2015)	anus swabs of HLC
GC82	S	S	S	S	S			ST92	+	Hubei (2015)	anus swabs of HLC
JS92	S	S	R	S	R			ST92	+	Hubei (2013)	anus swabs of HLC
JS104	S	S	S	S	R			ST92	+	Hubei (2013)	anus swabs of HLC
JS106	S	S	S	S	R			ST92	+	Hubei (2013)	anus swabs of HLC
JS107	S	S	S	I	R			ST92	+	Hubei (2013)	anus swabs of HLC
DY153	S	S	S	I	R			ST92	–	Hubei (2016)	anus swabs of HLC

^a^*CIP*, Ciprofloxacin, *OFL*, *Ofloxacin*, *ENR*, Enrofloxacin, *NOR*, Norfloxacin, *NA*, Nalidixic acid, *R* resistant, *I* intermediate, *S* susceptible

^b^Cutoff OD value = 0.21; “-”: non-biofilm producer (OD ≤ 0.21); “+”: weak biofilm producer (0.21 < OD ≤ 0.42); “++”: strong biofilm producer (OD > 0.42)

^c^*DC* diseased chicken, *HLC* healthy-looking chicken

MLST detected only two STs. Of these, 27 strains were ST-92, accounting for 90.0% of all *S.* Pullorum strains in this study (27/30), and the remaining three strains were ST-2151. Only one loci (hemD) was different between strains of ST-92 and ST-2151. G296C and A510G nucleotide substitutions were found in the *hemD* gene in strains of ST-2151 and this was not the case in strains of ST92. An additional file shows this in more detail (see Additional file 1). All the three strains of ST-2151 were isolated from chicken farms with serious outbreaks of PD.

### Biofilm-forming abilities

The ODc to define biofilm producer was OD_630nm_ = 0.210. Based on the OD_630nm_, 30 *S*. Pullorum isolates were classified into two groups. Fourteen isolates (46.7%) were identified as non-biofilm producers (OD_630_ ≤ 0.210), while 16 isolates (53.3%) were weak biofilm producers with OD_630nm_ ranging from 0.217–0.259, and there were no strong biofilm producers (OD_630nm_ > 0.420). In contrast, the OD_630nm_ of the *S.* Enteritidis reference strain was 0.441, classifying this as a strong biofilm producer. Among 18 isolates from chickens with from PD, eight were weak biofilm producers, and among 12 isolates from healthy-looking chickens, eight were weak biofilm producers. The fisher test showed that the positive biofilm rates between these two sources were not significant correlated (*p* > 0.05).

### Quinolones susceptibility testing

**Fig. 1:**
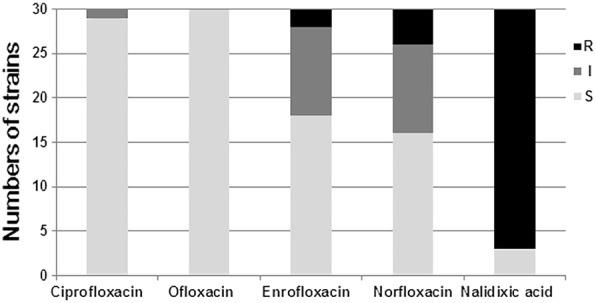
Quinolones resistance of *S*. pullorum isolates. R: resistant; I: intermediate; S: susceptible

As shown in Table 1 and Fig. 1, all of the *S.* pullorum isolates (100%) were susceptible to ofloxacin, and 29 strains (96.7%) were also susceptible to ciprofloxacin with the exception of one that was intermediate. Two strains (6.7%) were resistant to enrofloxacin, ten (33.3%) were intermediate, and 18 (60.0%) were susceptible for this antibiotic. Four strains (13.3%) were resistant to norfloxacin, 10 (33.3%) were intermediate, while 16 (53.3%) were susceptible. In contrast, 27 strains (90.0%) were resistant to nalidixic acid, which was a significantly higher resistance rate than resistance rates to fluoroquinolones (*p* < 0.05). Only three strains were susceptible to all of the tested (fluoro)quinolones. Three strains typed as ST-2151 were only susceptible to ofloxacin and ciprofloxacin. The correlation between biofilm-forming ability and quinolones susceptibility was further analyzed. Although the proportions of quinolones susceptible isolates of negative biofilm strains were higher than in weak biofilm strains, there was no significant correlation between biofilm and quinolones susceptibility in our study (Table 2).

**Table 2:** Correlation between biofilm-forming ability and quinolones susceptibility

Biofilm-forming abilites	Numbers of susceptible strains				
	Ciprofloxacin	Ofloxacin	Enrofloxacin	Norfloxacin	Nalidixic acid
Weak biofilm (*n* = 16)	15/16	16/16	8/16	7/16	1/16
None biofilm (*n* = 14)	14/14	14/14	10/14	9/14	2/14
*p* value	–	–	0.284	0.299	0.586

### Presence of PMQR and mutations in QRDR

Asp87Gly substitution in GyrA was found in three strains (3/30, 10%), and one of these three strains, WX46, had a Leu451Ile substitution. An additional file shows this in more detail (see Additional file 2). These three strains were the only three strains of ST-2151 in our study. The correlation between sequence type and Asp87Gly substitution was calculated by fisher test, and showed *p* < 0.01. Amino acid substitutions in topoisomerase *parC* and *parE* were not found. In addition, none of the *S.* Pullorum isolates had the *qnrA*, *qnrB*, *qnrC*, *qnrD*, *qnrS,* and *AAC-Ib* genes, and integrase and intergron were also not found in these isolates.

## Discussion

In recent years, *S*. Pullorum has been eradicated in the commercial poultry flocks in most of the developed countries. However, in some developing countries, including China, PD is still a serious problem in the poultry industry [4]. Investigating the genetic characterization of *S*. Pullorum will help to better understand the prevalence of this poultry pathogen. Considering that MLST has the advantage to compare the results across various laboratories, MLST was used to analyze the genetic diversity of *S*. Pullorum in this study. Previous studies showed that STs strongly correlate with serovars [29, 30]. Our results found that 27 out of 30 *S*. Pullorum strains were ST-92, which was consistent with previous reports [17]. This result suggested that ST-92 was the main genotype of *S*. Pullorum. In the study by Liu et al. of 17 tested *S*. Pullorum, other than strains of ST-92, one was ST-11, which was relatively similar to the sequence type of ST-92 [8]. In our study, three strains of ST-2151 were found, with only two substitutions in the *hemD* gene, and therefore we infered that ST-2151 was derived from microevolution of ST-92. To the best of our knowledge, this is the first report on ST-2151. *S*. Pullorum strains are characterized by D serogroup and the same O-antigens. It was shown, however, that a few *S*. Pullorum strains are slightly variable in O-antigens, resulting in variants and an intermediate type [31]. Whether there is a relationship between different STs and variation in antigens in *S*. Pullorum is unknown.

Gong et al. found that the fluoroquinolone resistance rates of *S*. Pullorum in China have strongly increased in recent years [32]. In this study, we tested the susceptibility of *S*. Pullorum isolates to five (fluoro)quinolones agents, including the first generation of quinolines, nalidixic acid, and four fluoroquinolones agents, which are currently widely used in the poultry industry. A high resistance rate to nalidixic acid was found, but the resistance rates to fluoroquinolones were relatively low, with no resistance to ciprofloxacin and ofloxacin in our test. Interestingly, the *S*. Pullorum isolates from one collection of samples showed different susceptibilities to tested (fluoro)quinolones agents. For example, strains GC81 and GC82 were susceptible to all of the tested agents, but strain GC80 was only susceptible to ofloxacin and norfloxacin.

As previously reported, PMQR and amino acid substitutions in QRDR, which widely exist in resistant *Salmonella*, can result in different levels of resistance to fluoroquinolones [11]. In some other pathogens, such as *Campylobacter*, substitutions in *gyrA* are very common, resulting in high resistance to fluoroquinolones [33]. Compared to the presence in some other bacterial pathogens and other serovars of *Salmonella*, no PMQR and a lower amount of mutations in QRDR were found in our tested *S*. Pullorum isolates. Mutations in QRDR were only present in the three strains of ST-2151, suggesting a potential correlation between microevolution and a resistant mutation. However, the strains with mutations in QRDR were also susceptible to ciprofloxacin and ofloxacin, which suggested that mutations in QRDR do not completely determine the susceptibility of *S*. Pullorum to fluoroquinolones. A number of isolates without mutations in QRDR were also resistance to (fluoro)quinolones, which suggested that more mechanisms were involved in (fluoro)quinolones resistance, such as efflux activity [34].

Compared with most other serovars of *Salmonella*, more than 50% of isolates from chickens resistant to fluoroquinolones [35, 36], resistance rates to fluoroquinolones were low in our tested *S*. Pullorum isolates. As previously reported, biofilm-forming abilities are positively correlated with antibiotic resistance [23]. However, a correlation between biofilm-forming ability and quinolones susceptibility in our tested isolates was not found. It might be due to the fact that the biofilm-forming abilities were extremely weak in our tested *S*. Pullorum isolates, and additionally, weak biofilm-forming abilities might also be an important reason of low resistance rates of *S*. Pullorum isolates. Formation of biofilms can protect bacteria against antibiotics by limiting penetration or forming specialized persistent cells. In our study, 14 isolates were non-biofilm producers, while the remaining 16 isolates could only form very weak biofilm (OD_630_ ranging from 0.217–0.259). In *Salmonella*, flagella are cell surface appendages involved in a number of bacterial behaviors, such as motility, adhesion, and biofilm formation [37]. However, *S*. Pullorum does not express the flagellum proteins [38]. Absence of flagella reduces the adhesive capacity of *S*. Pullorum and can lead to weak biofilm-forming abilities.

## Conclusions

In this study, a high resistance rate to the first generation of quinolines, nalidixic acid, but low resistance rates to fluoroquinolones agents were found in *S*. Pullorum isolates. In addition, weak biofilm-forming abilities were detected, which might explain the low fluoroquinolones resistance rates of *S*. Pullorum isolates. We found that ST-92 was the dominating genotype, and identified the new sequence type ST-2151, which showed potential correlation with Asp87Gly substitution in the QRDR of GyrA. We believe that the characterization of these *S*. Pullorum isolates will be helpful to develop prevention and control strategies.

## Additional files

Additional file 1: **Table S1.** The details of MLST assays of S. Pullorum isolates in this study. The sequence types, allele numbers and the sequences of seven housekeeping genes used for MLST assays were listed. (DOCX 19 kb)

Additional file 2: The details of Amino acid mutation in GyrA and GyrB in S. Pullorum isolates in this study. (a) The amino acid sequence in GyrA from 52 to 152 (QRDR) in wild type and mutant. (b) The amino acid sequence in GyrB from 359 to 507 in wild type and mutant. (DOCX 16 kb)

## Abbreviations

MLST: Multilocus Sequence Typing
PD: Pollorum disease
PMQR: Plasmid-mediated quinolone resistance
QRDR: Quinolone resistance-determining regions
STs: Sequence types

## References

[1] Shivaprasad HL (2000). Fowl typhoid and pullorum disease. Rev Sci Tech.

[2] Liu GR, Rahn A, Liu WQ, Sanderson KE, Johnston RN, Liu SL (2002). The evolving genome of Salmonella enterica serovar Pullorum. J Bacteriol.

[3] Wunderwald C, Hoop RK (2002). Serological monitoring of 40 Swiss fancy breed poultry flocks. Avian Pathol.

[4] Xie X, Hu Y, Xu Y, Yin K, Li Y, Chen Y, Xia J, Xu L, Liu Z, Geng S (2017). Genetic analysis of Salmonella enterica serovar Gallinarum biovar Pullorum based on characterization and evolution of CRISPR sequence. Vet Microbiol.

[5] Davidson RM (2002). Control and eradication of animal diseases in New Zealand. N Z Vet J.

[6] Eriksson H, Soderlund R, Ernholm L, Melin L, Jansson DS (2018). Diagnostics, epidemiological observations and genomic subtyping in an outbreak of pullorum disease in non-commercial chickens. Vet Microbiol.

[7] Gong J, Zhang J, Xu M, Zhu C, Yu Y, Liu X, Kelly P, Xu B, Wang C (2014). Prevalence and fimbrial genotype distribution of poultry Salmonella isolates in China (2006 to 2012). Appl Environ Microbiol.

[8] Liu WB, Liu B, Zhu XN, Yu SJ, Shi XM (2011). Diversity of Salmonella isolates using serotyping and multilocus sequence typing. Food Microbiol.

[9] Zhang T, Luo Q, Chen Y, Li T, Wen G, Zhang R, Luo L, Lu Q, Ai D, Wang H (2016). Molecular epidemiology, virulence determinants and antimicrobial resistance of campylobacter spreading in retail chicken meat in Central China. Gut pathogens.

[10] Nhung NT, Chansiripornchai N, Carrique-Mas JJ (2017). Antimicrobial resistance in bacterial poultry pathogens: a review. Front Vet Sci.

[11] Wasyl D, Hoszowski A, Zajac M (2014). Prevalence and characterisation of quinolone resistance mechanisms in Salmonella spp. Vet Microbiol.

[12] de Jong A, Smet A, Ludwig C, Stephan B, De Graef E, Vanrobaeys M, Haesebrouck F (2014). Antimicrobial susceptibility of Salmonella isolates from healthy pigs and chickens (2008-2011). Vet Microbiol.

[13] Pan Z, Wang X, Zhang X, Geng S, Chen X, Pan W, Cong Q, Liu X, Jiao X, Liu X (2009). Changes in antimicrobial resistance among Salmonella enterica subspecies enterica serovar Pullorum isolates in China from 1962 to 2007. Vet Microbiol.

[14] Seminati C, Mejia W, Mateu E, Martin M (2005). Mutations in the quinolone-resistance determining region (QRDR) of Salmonella strains isolated from pigs in Spain. Vet Microbiol.

[15] Ferrari R, Galiana A, Cremades R, Rodriguez JC, Magnani M, Tognim MC, Oliveira TC, Royo G (2013). Plasmid-mediated quinolone resistance (PMQR) and mutations in the topoisomerase genes of Salmonella enterica strains from Brazil. Braz J Microbiol.

[16] Lin D, Chen K, Wai-Chi Chan E, Chen S (2015). Increasing prevalence of ciprofloxacin-resistant food-borne Salmonella strains harboring multiple PMQR elements but not target gene mutations. Sci Rep.

[17] Maka L, Popowska M (2016). Antimicrobial resistance of Salmonella spp. isolated from food. Roczniki Panstwowego Zakladu Higieny.

[18] Alzwghaibi AB, Yahyaraeyat R, Fasaei BN, Langeroudi AG, Salehi TZ (2018). Rapid molecular identification and differentiation of common Salmonella serovars isolated from poultry, domestic animals and foodstuff using multiplex PCR assay. Arch Microbiol.

[19] Ibrahim HM, El-Moaty DA, Ahmed HA, El-Enbaawy MI (2016). Phenotypic and genotypic characterization of locally isolated Salmonella strains used in preparation of Salmonella antigens in Egypt. Vet World.

[20] Kang MS, Kwon YK, Jung BY, Kim A, Lee KM, An BK, Song EA, Kwon JH, Chung GS (2011). Differential identification of Salmonella enterica subsp. enterica serovar Gallinarum biovars Gallinarum and Pullorum based on polymorphic regions of glgC and speC genes. Vet Microbiol.

[21] Kotetishvili M, Stine OC, Kreger A, Morris JG, Sulakvelidze A (2002). Multilocus sequence typing for characterization of clinical and environmental salmonella strains. J Clin Microbiol.

[22] Brown HL, Reuter M, Salt LJ, Cross KL, Betts RP, van Vliet AH (2014). Chicken juice enhances surface attachment and biofilm formation of campylobacter jejuni. Appl Environ Microbiol.

[23] Zhang T, Dong J, Cheng Y, Lu Q, Luo Q, Wen G, Liu G, Shao H (2017). Genotypic diversity, antimicrobial resistance and biofilm-forming abilities of campylobacter isolated from chicken in Central China. Gut Pathogens.

[24] Yi K, Rasmussen AW, Gudlavalleti SK, Stephens DS, Stojiljkovic I (2004). Biofilm formation by Neisseria meningitidis. Infect Immun.

[25] Jin H, Zhou R, Kang M, Luo R, Cai X, Chen H (2006). Biofilm formation by field isolates and reference strains of Haemophilus parasuis. Vet Microbiol.

[26] Institute CaLS: Performance Standards for Antimicrobial Disk and Dilution Susceptibility Tests for Bacteria Isolated from Animals, approved Standard, Second ed. M31-A2, Wayne, PA.; 2002.

[27] Campioni F, Souza RA, Martins VV, Stehling EG, Bergamini AMM, Falcao JP (2017). Prevalence of gyrA mutations in Nalidixic acid-resistant strains of Salmonella Enteritidis isolated from humans, food, chickens, and the farm environment in Brazil. Microb Drug Resist.

[28] Kim J, Han X, Bae J, Chui L, Louie M, Finley R, Mulvey MR, Ferrato CJ, Jeon B (2016). Prevalence of plasmid-mediated quinolone resistance (PMQR) genes in non-typhoidal Salmonella strains with resistance and reduced susceptibility to fluoroquinolones from human clinical cases in Alberta, Canada, 2009-13. J Antimicrob Chemother.

[29] Achtman M, Hale J, Murphy RA, Boyd EF, Porwollik S (2013). Population structures in the SARA and SARB reference collections of Salmonella enterica according to MLST, MLEE and microarray hybridization. Infect Genet Evol.

[30] Zou QH, Li RQ, Liu GR, Liu SL (2016). Genotyping of Salmonella with lineage-specific genes: correlation with serotyping. Int J Infect Dis.

[31] Shi C, Singh P, Ranieri ML, Wiedmann M, Moreno Switt AI (2015). Molecular methods for serovar determination of Salmonella. Crit Rev Microbiol.

[32] Gong J, Xu M, Zhu C, Miao J, Liu X, Xu B, Zhang J, Yu Y, Jia X (2013). Antimicrobial resistance, presence of integrons and biofilm formation of Salmonella Pullorum isolates from eastern China (1962-2010). Avian Pathol.

[33] Zhang T, Cheng Y, Luo Q, Lu Q, Dong J, Zhang R, Wen G, Wang H, Luo L, Wang H, et al. Correlation between gyrA and CmeR box polymorphism and fluoroquinolone resistance in campylobacter jejuni isolates in China. Antimicrob Agents Chemother. 2017;61(7). 10.1128/AAC.00422-17 PMC5487682 28438942

[34] Gunell M, Webber MA, Kotilainen P, Lilly AJ, Caddick JM, Jalava J, Huovinen P, Siitonen A, Hakanen AJ, Piddock LJ (2009). Mechanisms of resistance in nontyphoidal Salmonella enterica strains exhibiting a nonclassical quinolone resistance phenotype. Antimicrob Agents Chemother.

[35] Wang Y, Zhang A, Yang Y, Lei C, Jiang W, Liu B, Shi H, Kong L, Cheng G, Zhang X (2017). Emergence of Salmonella enterica serovar Indiana and California isolates with concurrent resistance to cefotaxime, amikacin and ciprofloxacin from chickens in China. Int J Food Microbiol.

[36] Nhung NT, Van NTB, Cuong NV, Duong TTQ, Nhat TT, Hang TTT, Nhi NTH, Kiet BT, Hien VB, Ngoc PT (2018). Antimicrobial residues and resistance against critically important antimicrobials in non-typhoidal Salmonella from meat sold at wet markets and supermarkets in Vietnam. Int J Food Microbiol.

[37] Yang X, Thornburg T, Suo Z, Jun S, Robison A, Li J, Lim T, Cao L, Hoyt T, Avci R (2012). Flagella overexpression attenuates Salmonella pathogenesis. PLoS One.

[38] Thomson NR, Clayton DJ, Windhorst D, Vernikos G, Davidson S, Churcher C, Quail MA, Stevens M, Jones MA, Watson M (2008). Comparative genome analysis of Salmonella Enteritidis PT4 and Salmonella Gallinarum 287/91 provides insights into evolutionary and host adaptation pathways. Genome Res.

