# Supplementary material for: Quinolone resistance phenotype and genetic characterization of Salmonella enterica serovar Pullorum isolates in China, during 2011 to 2016
Source: BMC Microbiol. 2018 Dec 27;18:225. doi: 10.1186/s12866-018-1368-4 (PMC6307136; doi:10.1186/s12866-018-1368-4)
Supplement: Supplementary file 1 — Table S1. The details of MLST assays of S. Pullorum isolates in this study. The sequence types, allele numbers and the sequences of seven housekeeping genes used for MLST assays were listed. (DOCX 19 kb) [file 12866_2018_1368_MOESM1_ESM.docx]

Additional file 1

Table S1. The allele numbers of seven housekeeping genes in ST92 and ST2151

| Sequence Type | *aroC* | *dnaN* | *hemD* | *hisD* | *purE* | *sucA* | *thrA* |
| --- | --- | --- | --- | --- | --- | --- | --- |
| ST92 | 5 | 2 | 3 | 7 | 31 | 41 | 11 |
| ST2151 | 5 | 2 | 361 | 7 | 31 | 41 | 11 |

The MLST were analyzed in the Web (<http://enterobase.warwick.ac.uk/species/senterica/allele_st_search>).

The sequences of the seven housekeeping genes of ST92 were listed below.

***aroC* (allele numbers: 5)**

GTTTTTCGTCCGGGACACGCGGATTACACCTATGAGCAGAAATACGGCCTGCGCGATTACCGTGGCGGTGGACGTTCTTCCGCGCGTGAAACCGCGATGCGCGTAGCGGCAGGGGCGATCGCCAAGAAATACCTGGCGGAAAAGTTCGGCATCGAAATCCGCGGCTGCCTGACCCAGATGGGCGATATTCCGCTGGAGATTAAAGACTGGCGTCAGGTTGAGCTTAATCCGTTCTTTTGTCCCGATGCGGACAAACTTGACGCGCTGGACGAACTGATGCGCGCGCTGAAAAAAGAGGGCGACTCCATCGGCGCGAAAGTGACGGTGATGGCGAGCGGCGTGCCGGCAGGGCTTGGCGAACCGGTTTTTGACCGACTGGATGCGGACATCGCCCATGCGCTGATGAGCATCAATGCGGTGAAAGGCGTGGAGATCGGCGAAGGATTTAACGTGGTGGCGCTGCGCGGCAGCCAGAATCGCGATGAAATCACGGCGCAGGGT

***dnaN* (allele numbers: 2)**

ATGGAGATGGTCGCGCGCGTTACGCTTTCTCAGCCGCATGAGCCGGGCGCCACTACCGTGCCGGCGCGGAAATTCTTTGATATCTGCCGCGGCCTGCCGGAGGGCGCGGAGATTGCCGTTCAGTTGGAAGGCGATCGGATGCTGGTGCGTTCTGGCCGTAGCCGCTTCTCGCTGTCTACGCTGCCTGCCGCCGATTTCCCGAATCTTGACGACTGGCAAAGCGAAGTTGAATTTACGCTGCCGCAGGCCACGATGAAGCGCCTGATTGAAGCGACCCAGTTTTCGATGGCTCATCAGGATGTGCGCTACTACTTAAACGGTATGCTGTTTGAAACGGAAGGTAGCGAACTGCGCACTGTCGCGACCGACGGCCACCGCCTGGCGGTGTGCTCAATGCCGCTGGAAGCGTCTTTACCCAGCCACTCGGTGATTGTGCCGCGTAAAGGCGTGATTGAACTGATGCGTATGCTCGACGGCGGTGAAAACCCGCTGCGCGTGCAG

***hemD* (allele numbers: 3)**

GCGACACTGACGGAAAACGATCTGGTTTTTGCCCTTTCACAGCACGCCGTCGCCTTTGCTCACGCCCAGCTCCAGCGGGATGGCCGAAACTGGCCTGCGTCGCCGCGCTATTTCGCGATTGGCCGCACCACGGCGCTCGCCCTTCATACCGTTAGCGGGTTCGATATTCGTTATCCATTGGATCGGGAAATCAGCGAAGCCTTGCTACAATTACCTGAATTACAAAATATTGCGGGCAAACGCGCGCTGATTTTGCGTGGCAATGGCGGCCGCGAACTGCTGGGCGAAACCCTGACAGCTCGCGGAGCCGAAGTCAGTTTTTGTGAATGTTATCAACGATGTGCGAAACATTACGATGGCGCGGAAGAAGCGATGCGCTGGCATACTCGCGGCGTAACAACGCTTGTTGTTACCAGCGGCGAGATGTTGCAA

***hisD* (allele numbers: 7)**  ATTGCGGGATGTCAGAACGTGGTTCTGTGCTCGCCGCCGCCCATCGCTGATGAAATCCTCTATGCGGCGCAACTGTGTGGCGTGCAGGAAATCTTTAACGTCGGCGGCGCGCAGGCGATTGCCGCTCTGGCCTTCGGCAGCGAGTCCGTACCGAAAGTGGATAAAATTTTTGGCCCCGGCAACGCCTTTGTAACCGAAGCCAAACGTCAGGTCAGCCAACGCCTCGACGGCGCGGCTATCGATATGCCAGCCGGGCCGTCTGAAGTACTGGTGATCGCCGACAGCGGCGCAACACCGGATTTCGTCGCTTCTGACCTGCTCTCCCAGGCTGAGCACGGTCCGGATTCGCAGGTGATTCTGCTGACGCCTGATGCTGACATTGCCTGCAAGGTGGCGGAGGCGGTAGAACGTCAACTGGCAGAACTGCCGCGCGCGGACACCGCCAGGCAGGCCCTGAGCGCCAGTCGTCTGATTGTGACCAAAGATTTAGCGCAGTGCGTC

***purE* (allele numbers: 31)** AGCGACTGGGCTACCATGCAATTCGCCGCCGAAATTTTTGAAATTCTGGATGTCCCGCACCATGTAGAAGTGGTTTCCGCCCATCGCACCCCCGATAAACTGTTCAGCTTCGCCGAAACGGCGGAAGAGAACGGATATCAAGTGATTATTGCCGGCGCGGGCGGCGCGGCGCACCTGCCGGGAATGATTGCGGCAAAAACGCTGGTCCCGGTACTCGGCGTGCCGGTACAAAGCGCTGCGCTAAGCGGCGTGGACAGCCTCTACTCCATTGTGCAGATGCCGCGCGGCATTCCGGTGGGTACGCTGGCGATCGGTAAAGCCGGTGCCGCTAACGCCGCCCTGCTCGCCGCGCAGATTCTGGCGCAACACGACGCGGAACTGCATCAGCGCATTGCCGAC

***sucA* (allele numbers: 41)**

AAACGCTTCCTGAACGAACTGACCGCCGCTGAAGGGCTAGAACGTTATCTGGGTGCCAAATTCCCGGGTGCGAAACGTTTCTCGCTCGAGGGGGGAGATGCGCTGATACCCATGCTGAAAGAGATGGTTCGCCATGCGGGTAACAGCGGCACTCGCGAAGTGGTGCTGGGGATGGCGCACCGCGGTCGCCTGAACGTGCTGATCAACGTACTGGGTAAAAAACCGCAGGATCTGTTCGACGAATTTGCCGGTAAGCATAAAGAACATCTGGGTACCGGCGACGTGAAGTATCACATGGGCTTCTCGTCAGATATCGAAACCGAAGGCGGTCTGGTTCACCTGGCGCTGGCGTTTAACCCATCGCATCTGGAAATTGTGAGCCCGGTGGTGATGGGCTCCGTGCGCGCCCGTCTGGACAGACTGGACGAACCGAGCAGCAACAAAGTGTTGCCGATCACTATTCACGGCGACGCCGCGGTGACCGGCCAGGGCGTGGTTCAG

***thrA* (allele numbers: 11)** GTGCTGGGCCGTAATGGTTCCGACTATTCCGCCGCCGTGCTGGCCGCCTGTTTACGCGCTGACTGCTGTGAAATCTGGACTGACGTCGATGGCGTGTATACCTGTGACCCGCGCCAGGTGCCGGACGCCAGGCTGCTGAAATCGATGTCCTACCAGGAAGCGATGGAACTCTCTTACTTCGGCGCCAAAGTTCTTCACCCTCGCACCATTACGCCCATCGCCCAGTTCCAGATCCCCTGTCTGATTAAAAATACCGGTAATCCGCAGGCGCCAGGAACGCTGATCGGCGCGTCCAGCGACGATGATAACCTGCCGGTTAAAGGGATCTCTAACCTTAACAACATGGCGATGTTTAGCGTCTCCGGCCCGGGAATGAAAGGGATGATTGGGATGGCGGCGCGTGTTTTCGCCGCCATGTCTCGCGCCGGGATCTCGGTGGTGCTCATTACCCAGTCCTCCTCTGAGTACAGCATCAGCTTCTGTGTGCCGCAGAGTGACTGC

The only difference of ST2151 and ST92 was the gene hemD, and the sequence of hemD in ST2151 was listed below.

***hemD* (allele numbers: 361)**

GCGACACTGACGGAAAACGATCTGGTTTTTGCCCTTTCACAGCACGCCGTCGCCTTTGCTCACGCCCAGCTCCAGCGGGATGGCCGAAACTGGCCTGCGTCGCCGCGCTATTTCGCGATTGGCCGCACCACGGCGCTCGCCCTTCATACCGTTAACGGGTTCGATATTCGTTATCCATTGGATCGGGAAATCAGCGAAGCCTTGCTACAATTACCTGAATTACAAAATATTGCGGGCAAACGCGCGCTGATTTTGCGTGGCAATGGCGGCCGCGAACTGCTGGGCGAAACCCTGACAGCTCGCGGAGCCGAAGTCAGTTTTTGTGAATGTTATCAACGATGTGCGAAACATTACGATGGCGCGGAAGAGGCGATGCGCTGGCATACTCGCGGCGTAACAACGCTTGTTGTTACCAGCGGCGAGATGTTGCAA

The point substitute were marked with underline.
