# Supplementary material for: Quinolone resistance phenotype and genetic characterization of Salmonella enterica serovar Pullorum isolates in China, during 2011 to 2016
Source: BMC Microbiol. 2018 Dec 27;18:225. doi: 10.1186/s12866-018-1368-4 (PMC6307136; doi:10.1186/s12866-018-1368-4)
Supplement: Supplementary file 2 — The details of Amino acid mutation in GyrA and GyrB in S. Pullorum isolates in this study. (a) The amino acid sequence in GyrA from 52 to 152 (QRDR) in wild type and mutant. (b) The amino acid sequence in GyrB from 359 to 507 in wild type and mutant. (DOCX 16 kb) [file 12866_2018_1368_MOESM2_ESM.docx]

Additional file 2. The details of Amino acid mutation in GyrA and GyrB in S. Pullorum isolates in this study.

(a) The amino acid sequence in GyrA from 52 to 152 (QRDR) in wild type and mutant.

Original sequences in wild type:

MNVLGNDWNKAYKKSARVVGDVIGKYHPHGDSAVYDTIVRMAQPFSLRYMLVDGQGNFGSIDGDSAAAMRYTEIRLAKIAHELMADLEKETVDFVDNYDGT

Asp87Gly substitution in WX46、WX47 and WH59 strains:

MNVLGNDWNKAYKKSARVVGDVIGKYHPHGDSAVY**G**TIVRMAQPFSLRYMLVDGQGNFGSIDGDSAAAMRYTEIRLAKIAHELMADLEKETVDFVDNYDGT

(b) The amino acid sequence in GyrB from 359 to 507 in wild type and mutant.

Original sequence in wild type:

EYLLENPSDAKIVVGKIIDAARAREAARRAREMTRRKGALDLAGLPGKLADCQERDPALSELYLVEGDSAGGSAKQGRNRKNQAILPLKGKILNVEKARFDKMLSSQEVATLITALGCGIGRDEYNPDKLRYHSIIIMTDADVDGSHIR

Leu451Ile substitution in WX46 strian:

EYLLENPSDAKIVVGKIIDAARAREAARRAREMTRRKGALDLAGLPGKLADCQERDPALSELYLVEGDSAGGSAKQGRNRKNQAILPLKGKI**I**NVEKARFDKMLSSQEVATLITALGCGIGRDEYNPDKLRYHSIIIMTDADVDGSHIR
